# Evaluating Primary Blast Effects *In Vitro*

**DOI:** 10.3791/55618

**Published:** 2017-09-18

**Authors:** Niall J. Logan, Hari Arora, Claire A. Higgins

**Affiliations:** ^1^Department of Bioengineering, Imperial College London

**Keywords:** Bioengineering, Issue 127, Shock wave, blast physics, shock tube, viability, cell culture, live and dead cell analysis

## Abstract

Exposure to blast events can cause severe trauma to vital organs such as the lungs, ears, and brain. Understanding the mechanisms behind such blast-induced injuries is of great importance considering the recent trend towards the use of explosives in modern warfare and terrorist-related incidents. To fully understand blast-induced injury, we must first be able to replicate such blast events in a controlled environment using a reproducible method. In this technique using shock tube equipment, shock waves at a range of pressures can be propagated over live cells grown in 2D, and markers of cell viability can be immediately analyzed using a redox indicator assay and the fluorescent imaging of live and dead cells. This method demonstrated that increasing the peak blast overpressure to 127 kPa can stimulate a significant drop in cell viability when compared to untreated controls. Test samples are not limited to adherent cells, but can include cell suspensions, whole-body and tissue samples, through minor modifications to the shock tube setup. Replicating the exact conditions that tissues and cells experience when exposed to a genuine blast event is difficult. Techniques such as the one presented in this article can help to define damage thresholds and identify the transcriptional and epigenetic changes within cells that arise from shock wave exposure.

**Figure:**
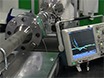


## Introduction

With the recent trend towards the use of improvised explosive devices in modern warfare and terrorist actions on civilians, understanding the effects of explosive events on the human body is of great importance. Injuries obtained through exposure to blast events can be deadly and lethal, with the physical processes of injury being divided into four categories. Primary injuries result from direct exposure to the blast wave, which interacts locally with the body in a compressive and subsequently expansive manner, causing the disruption of membranes and soft tissues^1^. Secondary injuries include blunt trauma or penetrative wounds caused by impact with low-mass objects propelled at high velocity by the blast wave. Tertiary injuries occur when the blast wave has sufficient energy to throw objects of high mass or individuals against objects. Lastly, quaternary blast injuries are defined by other miscellaneous injuries that do not fit the other categories, such as flash burns^2^. Following exposure to such blast events, primary injuries include traumatic brain injury^3^,^4^,^5^, heterotopic ossification^6^,^7^, blast lung injury^8^, loss of hearing^9^, and others^10^.

A commonly observed waveform from blast events is the Friedlander wave, representing a free-field, as opposed to an enclosed-space, explosion. The waveform consists of a blast front that can be defined as a sharp and rapid rise in positive pressure. This is immediately followed by a blast wind of air moving at high speed and a release wave that reduces the pressure to below atmospheric levels. A partial vacuum is left at the region of the initial explosion, which results in the slow backflow of air. The positive and negative phases of the wave (**Figure 1A**) result in the push-pull movement of the blast wave^1^. To help elucidate the mechanisms behind primary blast injuries, experimental models have been created to produce waveforms, such as the Friedlander wave, which cells and tissues will face when exposed to a genuine blast event. Current systems listed in the literature include shock tubes^11^,^12^,^13^,^14^,^15^,^16^,^17^, barochambers^18^,^19^, the Kolsky bar^20^, advanced blast simulators^21^, the Split Hopkinson pressure bar^22^, and the recreation of alternative blast events in a controlled environment using pentaerythritol tetranitrate^23^. Despite the wide range of models available, many variables influence the injury obtained from blast waves, including the pre-stress applied to, and the mechanical properties of, the individual cell types or tissues under evaluation^24^. While the study of tissue or organs can shed light on tissue deformation and gross morphological changes sustained as a result of blast events, analysis at the cellular level can uncover transcriptional and epigenetic changes influenced by the shock wave.

This methods article describes a technique to propagate shock waves at a range of pressures over live cells in a monolayer. This allows for the immediate characterization of cell viability, elucidating potential damage thresholds from shock waves. Furthermore, viable cells can be returned to standard culture conditions, and long-term biological effects from the blast event can be assessed. The protocol below describes two cell viability techniques that can be used on cells in culture.

**Figure:**
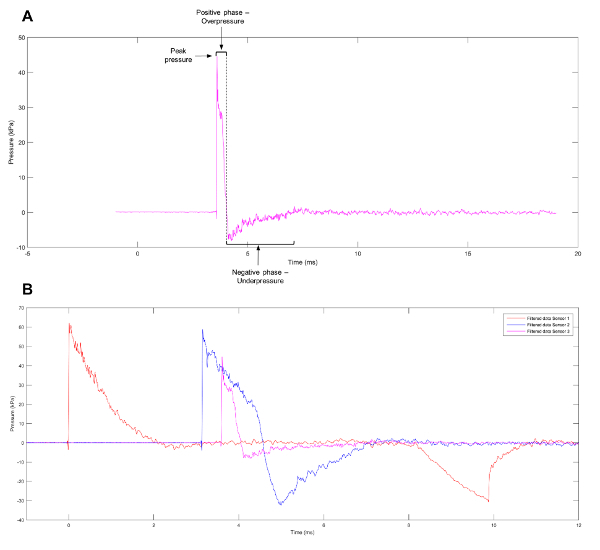

Figure 1:** Approximation of a Friedlander Wave.** (**A**) An approximation of a Friedlander wave observed at sensor 3 on the shock tube. (**B**) Representative data showing the different pressure profiles observed at sensors 1, 2, and 3 on the shock tube. Please click here to view a larger version of this figure.

## Protocol

### 1. Cell Culture and Sample Preparation

1. Obtain an appropriate cell line *(e.g.,* dermal papilla cells). NOTE: The current protocol has been designed for adherent cells, with rat dermal papilla fibroblasts isolated from vibrissae used as a model.
2. Culture the cells in a T-75 flask containing minimum essential medium (MEM) α supplemented with 10% fetal bovine serum and 1% penicillin streptomycin. Keep the cells at standard culture conditions of 37 °C and 5% CO_2_ in a humidified environment until they reach 80% confluence.
3. Aspirate the medium and wash the cells twice in Dulbecco's phosphate-buffered saline (PBS). Dissociate the cells from the flask by incubating them in 2 mL of trypsin/EDTA at standard culture conditions for 5 min. Briefly check to ensure that cells have successfully dissociated from the flask using a light/phase contrast microscope (with a 10X objective lens).
4. Add 4 mL of culture medium to neutralize the trypsinization reaction. Transfer the cell suspension to a 15 mL centrifuge tube.
5. Centrifuge at 200 x g for 4 min to pellet the cells.Slowly aspirate the supernatant, taking care not to dislodge the pellet.Re-suspend the pellet in 5 mL of medium.
6. Transfer a 20 µL aliquot of the cell suspension to a clean hemocytometer and count the cells using a microscope fitted with a 10X objective lens.
7. Prepare the cells for the blast by placing three 35 mm dishes inside a 90 mm dish. Label them appropriately. Add 2 mL of medium to each 35 mm dish and seed a total of 5 x 10^4 ^cells per dish, distributing the cells evenly around the dish. Incubate the experimental samples overnight in standard culture conditions to allow for adequate cell attachment before shock wave exposure. NOTE: For fluorescent imaging, cells must be seeded onto sterile glass coverslips.

### 2. Shock Tube Assembly

**Figure:**
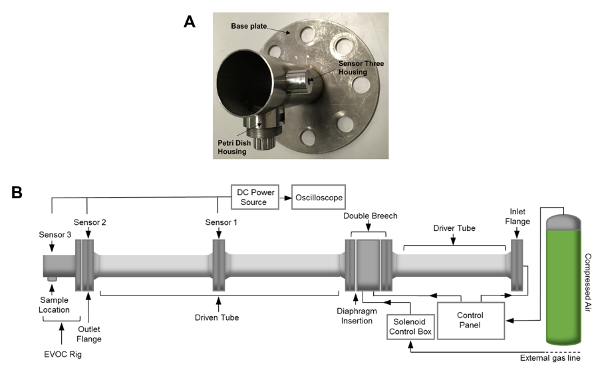

Figure 2**: EVOC Rig and Shock Tube Assembly.** (**A**) Image of the EVOC rig. (**B**) Schematic of the shock tube apparatus. Shock tube dimensions include an internal diameter of 59 mm and a total length, including the EVOC rig, of 4.13 m. The length of the driven tube and the EVOC rig totals 2.71 m. Please click here to view a larger version of this figure.

1. Wear steel-toe work boots and a laboratory coat during the assembly procedures.
2. Insert the two alignment bars through the two bolt holes found in the horizontal plane of the outlet flange. Place a nitrile-rubber gasket sheet over the end of the alignment bars so that it sits between the outlet flange and the *ex vivo* organ culture (EVOC) rig. NOTE: The EVOC rig is a custom-designed fixture intended for use with 35 mm Petri dishes (Figure 2A).
3. Attach the EVOC rig to the outlet flange of the shock tube by sliding the fixture over the alignment bars, ensuring that it is orientated correctly so that the section that holds the petri dish is located at its base.
4. Insert four M24 bolts and washers into the remaining holes. Position the nuts so that they touch the flange.
5. Tightly fasten the nuts and bolts sequentially in a diagonally symmetric fashion whilst visually checking that the EVOC rig and shock tube have successfully aligned.
6. Attach a pressure transducer to the EVOC rig and connect it to the current source using a sensor cable. Then, using a BNC cable, connect the current source to the oscilloscope.
7. Ensure that all release valves and flow controls on the shock tube are closed. Open the built-in external compressed air line and manually turn the pressure regulator clockwise to charge the solenoid to 2.5 bar.
8. Open the safety valve on the compressed gas bottle by turning it anticlockwise 180°.
9. Slowly turn the pressure regulator, located on top of the gas bottle, clockwise to increase the pressure to approximately 15 bar. NOTE: The setting point of this regulator should be slightly above the highest bursting pressure of the thickest diaphragm that is to be used in the experiment.
10. Prepare the diaphragms by cutting a 0.125 mm-thick plastic sheet into 10 cm x 10 cm squares. NOTE: The thickness of the diaphragm will correlate with the bursting pressure, altering the peak pressure of the shock wave (*i.e.,* a thicker diaphragm will result in a shock wave with a larger peak pressure; **Table 1**).

**Table:** 

**Mylar Diaphragm dimensions (cm)**	**Burst pressure (bar)**	**Sensor 3 pressure range (kPa)**
10 x 10 x 0.023	2 ± 0.2	47 ± 7.5
10 x 10 x 0.050	4 ± 0.2	72 ± 7.5
10 x 10 x 0.125	9.5 ± 0.5	127 ± 12.5

**Table 1: Burst Pressure Corresponding to Diaphragm Thickness and Peak Pressure.**

1. Create handles out of tape, fix them to the top and bottom of the diaphragm, and use them to carefully position the diaphragm next to the front small flange by the breech chamber. Ensure that the diaphragm is straight and correctly positioned before closing the breech.
2. Secure this using four M24 bolts and nuts. Tightly fasten the nuts and bolts sequentially in a diagonally symmetric fashion.

### 3. Preparation of Cells Just Prior to Shock Wave Exposure

1. Prepare a tissue laminar flow hood by performing ultraviolet sterilization. Clean it with a disinfectant solution and 70% ethanol. Gather all items needed for handling cells prior to/post-shock wave exposure and place them within the sterile hood. NOTE: This includes fresh medium, adhesive gas-permeable membranes, sterile scissors, pipettes, and pipette tips.
2. Transfer one 90 mm Petri dish containing three experimental samples from the incubator to the sterile hood.
3. Cut an adhesive gas-permeable membrane sheet (see the **Table of Materials**) into six squares, such that each is large enough to cover the top of one 35 mm Petri dish.
4. Aspirate the medium from the 35 mm Petri dish, place the lid to one side, and cover with two adhesive gas-permeable membranes sections, ensuring that there is a tight seal between the membrane and the edge of the dish.
5. Transfer the sample to the EVOC rig and secure it to the base of the fixture so that the top of the dish is aligned with the inner base of the shock tube.

### 4. Shock Tube Operation

1. Wear ear defenders, eye goggles, safety boots, and a laboratory coat when pressurizing the shock tube system.
2. Turn the flow control knob on the regulator clockwise, allowing gas to flow into the flexible hose connecting the compressed air cylinder to the shock tube.
3. At the bottom of the control panel, select either the "Double Breech" inlet for a short-duration shock wave or the "Driver Tube" inlet for an increased wave duration.
4. Switch on the DC power source and oscilloscope to acquire the waveform data. Set the oscilloscope sampling rate to 50.0 MS/s, with a record length of 1 M points. Set a range of 50 mV for firing at 2 bar and 100 mV for above 4 bar.
5. Turn on the safety lights using the switch on the wall opposite the control panel. NOTE: This tells other researchers not to enter the room when the shock tube is charging.
6. Turn on the switch on the solenoid control box to close the solenoid. NOTE: This safety feature closes a valve located on the double breech chamber, allowing for the system to be charged.
7. On the control panel, slowly open the flow control by turning the knob counterclockwise to gradually increase the pressure within the compression chamber. Monitor the pressure using the control panel display.
8. Once the diaphragm burst pressure is reached, the diaphragm will rupture and a "bang" will be heard. Quickly close the flow control by turning the knob. NOTE: The shock wave will travel down the tube over the cell sample and out the end of the tube.
9. Open the solenoid by turning off the switch on the solenoid control box and switch off the safety light (using the switch on the wall opposite the control panel).
10. Transfer the cell sample to the sterile hood, remove the adhesive gas-permeable membranes, and fill the dish with 2 mL of fresh growth medium. The cells can be used immediately for assessment, as described in step 5, or returned to the incubator for longer-term studies.

### 5. Cell Viability

1. Assess cell viability following shock wave exposure using the combination of a redox indicator assay and the fluorescent imaging of live and dead cells. NOTE: For fluorescent imaging, cells must be seeded onto sterile glass coverslips during section 1: Cell culture and sample preparation. These can be placed within the 35-mm petri dishes.
2. Transfer 200 µL of the redox indictor reagent (see the **Table of Materials**) to each experimental dish directly after refilling them with 2 mL of medium post-shock wave exposure. Incubate at standard culture conditions for 4 h.
3. For each dish, transfer (in a dark sterile hood) 2 x 100 µL aliquots to either a black or clear 96-well plate, depending on whether fluorescence or absorbance will be used to assess viability.
4. Transfer the 96-well plate to an appropriate plate reader fitted with the correct filters and read using excitation bands 530ex/590em for fluorescence or 570 nm combined with a 600-nm reference for absorbance. Export and save the data.
5. Analyze the data to identify any difference between shock wave-exposed samples and non-exposed controls, as this can indicate a drop in cell viability. NOTE: A negative control should also be included in the experimental design. Furthermore, the interpolation of a standard curve can be used to calculate cell numbers.
6. Aspirate the medium containing the redox indicator reagent. Replace it with 2 mL of fresh medium and incubate the cells at standard culture conditions for 24 h.
7. Repeat the above steps as necessary to obtain a second viability time point.
8. For the fluorescent imaging of live and dead cells, aspirate the medium and wash the cells twice in 1 mL of PBS. Transfer 100 µL of the complete reagent found in the imaging kit (see the **Table of Materials**) to each sample and incubate at room temperature protected from light for 15 min.
9. Aspirate the reagent and wash once using 1 mL of PBS. Using fine point forceps, carefully remove the coverslip from the 35 mm dish and place it face-down onto a glass microscopy slide.
10. Acquire images for each sample using a fluorescent microscope (10X objective lens, 0.50 numerical aperture) fitted with the correct excitation and emission filters (ex/em 488 nm/515 nm and 570 nm/602 nm). NOTE: Live cells will emit intense, uniform, green fluorescence. Dead or dying cells with a compromised cellular membrane will uptake the dead kit component, resulting in the emission of red fluorescence, found predominantly in the nuclear region of the cell.
11. Use image analysis software to either manually or automatically count the number of live and dead cells from each image.

## Representative Results

Using the method described above, cells grown in a monolayer were exposed in triplicate to shock waves generated using a shock tube (Figure 2B). Markers of cell viability were assessed. Using a redox indicator assay, it was found that the application of a 127 kPa shock wave was able to significantly reduce the viability of dermal papilla cells compared to controls after 24 h in culture (Figure 3). The application of a shock wave ≤72 kPa did not reduce viability. To support these observations, a fluorescent image assay capable of fluorescently labelling live or dead cells with green or red fluorophores, respectively, was used. The quantification of live and dead cells from 13 fluorescent images per biological replicate demonstrated a reduction in cell viability in those exposed to the 127 kPa shock wave when compared to the control (Figure 4). Statistical analysis was carried out using a one-way ANOVA followed by Tukey's multiple comparison test, with p <0.05 deemed statistically significant. The sample size per group totaled 9 for the redox indicator assay and 39 for the quantitative fluorescence imaging analysis.

**Figure:**
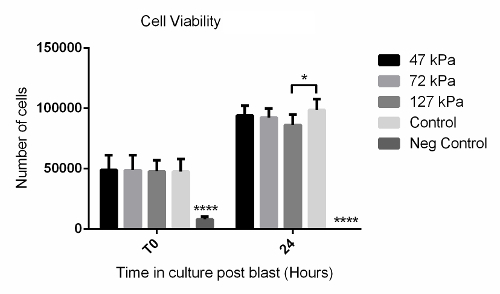

Figure 3**: Redox Indicator Assay Data Showing Time Course of Cell Viability After Shock Wave Exposure. **Significantly fewer cells were observed after 24 h in the 127-kPa shock wave-exposed group when compared to the untreated control. The negative control that consisted of a 30-s treatment with 2% disinfectant (see the Table of Materials) showed significantly fewer cells at both T0 and 24 h compared to all other groups. Each bar represents the mean ± 1 standard deviation (SD); biological replicates, N = 3; technical replicates, n = 3. **** = p <0.0001. * = p <0.05. Please click here to view a larger version of this figure.

**Figure:**
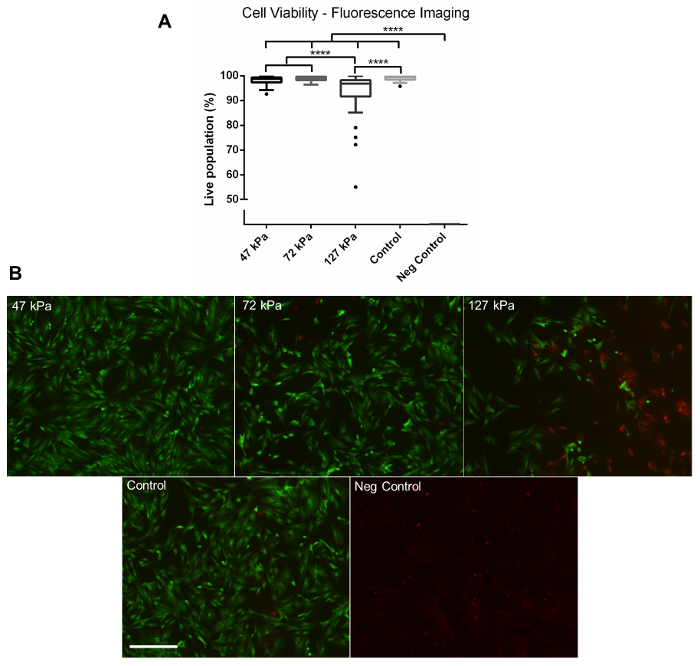

Figure 4**: Fluorescence Imaging.** (**A**) Quantitative data gathered from fluorescent images of live and dead cells captured using a fluorescence microscope at 24 h post-shock wave exposure. Significantly fewer viable cells were observed in the 127 kPa (mean = 93.42) shock wave-exposed sample compared to the 47 kPa (mean = 98.18), 72 kPa (mean = 98.87), and control groups (mean = 99.10). No viable cells were observed on the negative control group after 24 h (mean = 0). (**B**) Representative fluorescence images. Green shows live cells, whilst red shows dead cells. Each box plot shows the upper and lower quartiles with Tukey whiskers; biological replicates, N = 3; technical replicates, n = 13. **** = p <0.0001. Scale bar = 300 µm. Please click here to view a larger version of this figure.

## Discussion

Primary injuries obtained from exposure to blast events are not yet fully understood. Identifying and understanding the mechanisms that trigger blast-induced injuries, such as traumatic brain injury^3^,^4^ and heterotopic ossification^6^,^7^, are important first steps to developing effective methods of prophylaxis. To help achieve this goal, a number of experimental systems have been developed to replicate blast event exposure^11^,^12^,^13^,^14^,^18^,^19^. The technique described here uses shock tube equipment (Figure 2) capable of firing shock waves at a range of pressures at whole-body (*i.e., *rodent), tissue, or cell samples. The ability to load individual cell types rather than whole tissues gives the ability to parse out distinct cellular responses, as damage can occur concurrently via a range of mechanisms^1^,^2^. For example, to model traumatic brain injury, the assessment of individual cell types, such as neurons and astrocytes, can allow for the identification of cell-specific injury. Also, the whole-organ response can be assessed using brain tissue. Both the individual cell types and the tissue specimens have value and can give different information. It is also possible to alter the amount of air that is pressurized to generate the shock by selecting the double-breech or driver-tube inlet. This controls the duration of the shock wave. Another possibility is to change the diaphragm material and thickness to alter the peak pressure^25^.

Another factor to consider are interference end effects that can be present when the sample housing is located near the exit of the shock tube, such as that found on the EVOC rig described in the present system. Chandra* et al. *looked at blast wave profiles found on different locations on a compression-driven shock tube and found that the Friedlander waveform was best represented at a location deep within the shock tube^15^. Kuriakose* et al. *also studied secondary loading of the sample and found that the placement of an end plate at the end of the shock tube was able to eliminate unwanted reflected waves^16^. Considering the data found in these publications^15^,^16^, future modifications to improve the shock tube system described in this article could involve the placement of the EVOC rig at a deeper location within the driven tube or, alternatively, the inclusion of an end plate on the shock tube. Limitations of the described method could include the relatively low throughput of samples. A single user can operate the shock tube safely at an output of around 6-8 samples per hour. At present, the system is designed around the use of single 35-mm petri dishes. Therefore, larger experiments containing multiple groups and biological replicates can be difficult to achieve.

This methods article shows how the viability of adherent dermal papilla cells was affected by exposure to a single shock wave. A short-duration shock wave (<10 ms) of ≤72 kPa did not affect viability when compared to the control (Figure 3 and Figure 4). In contrast, a shock wave at 127 kPa stimulated a significant drop in viability at 24 h post-blast, as shown by both a redox indicator assay (Figure 3) and fluorescent image analysis (Figure 4). Miller* et al.* reported a similar reduction in cell viability in rat organotypic hippocampal slice cultures when cells were exposed to a either a 147 kPa or 278 kPa shock wave using an open-ended, helium-driven shock tube^14^. In contrast, VandeVord *et al.* reported that there was no effect on viability in rat astrocytes exposed to a short-duration overpressure of >200 kPa, although a barochamber was used rather than a shock tube^18^. It should be noted that the external pressure is reliant on the blast wave, although this creates complex stress waves within the body, therefore making the nature of the loading highly dependent upon the mechanical properties of the tissue or cell. Additional characterization studies of the cellular response to blast events is required. Furthermore, by assessing shock wave exposure at the cellular level, as shown in this technique, biological responses triggered from the injury, such as the perturbation of signaling pathways or epigenetic changes, can be identified and explored further.

In conclusion, this work describes the use of a stainless-steel shock tube and a modified EVOC rig to incorporate primary cell cultures. Shock waves at a range of pressures can be generated and propagated over live cells to replicate the effects that occur from exposure to a blast wave. This protocol demonstrates how to evaluate cell viability, but longer-term changes to individual cell types can also be studied. Going forward, we plan to assess the differential effects that complex shock waves can elicit in different cell types, with the aim of furthering our understanding of blast-induced primary injuries.

## Disclosures

The authors have no competing financial interests.
